# Identification of the core bacteria in rectums of diarrheic and non-diarrheic piglets

**DOI:** 10.1038/s41598-019-55328-y

**Published:** 2019-12-10

**Authors:** Jing Sun, Lei Du, XiaoLei Li, Hang Zhong, Yuchun Ding, Zuohua Liu, Liangpeng Ge

**Affiliations:** 1 Chongqing Academy of Animal Sciences, Chongqing, 402460 China; 2 Institute of Animal Genetics and Breeding, Sichuan Agricultural University, Chengdu, 611130 China; 3 Key Laboratory of Pig Industry Sciences, Ministry of Agriculture, Chongqing, 402460 China; 4 Chongqing Key Laboratory of Pig Industry Sciences, Chongqing, 402460 China

**Keywords:** Microbiome, Microbial communities

## Abstract

Porcine diarrhea is a global problem that leads to large economic losses of the porcine industry. There are numerous factors related to piglet diarrhea, and compelling evidence suggests that gut microbiota is vital to host health. However, the key bacterial differences between non-diarrheic and diarrheic piglets are not well understood. In the present study, a total of 85 commercial piglets at three pig farms in Sichuan Province and Chongqing Municipality, China were investigated. To accomplish this, anal swab samples were collected from piglets during the lactation (0–19 days old in this study), weaning (20–21 days old), and post-weaning periods (22–40 days), and fecal microbiota were assessed by 16S rRNA gene V4 region sequencing using the Illumina Miseq platform. We found age-related biomarker microbes in the fecal microbiota of diarrheic piglets. Specifically, the family *Enterobacteriaceae* was a biomarker of diarrheic piglets during lactation (cluster A, 7–12 days old), whereas the *Bacteroidales* family *S24–7* group was found to be a biomarker of diarrheic pigs during weaning (cluster B, 20–21 days old). Co-correlation network analysis revealed that the genus *Escherichia-Shigella* was the core component of diarrheic microbiota, while the genus *Prevotellacea UCG-003* was the key bacterium in non-diarrheic microbiota of piglets in Southwest China. Furthermore, changes in bacterial metabolic function between diarrheic piglets and non-diarrheic piglets were estimated by PICRUSt analysis, which revealed that the dominant functions of fecal microbes were membrane transport, carbohydrate metabolism, amino acid metabolism, and energy metabolism. Remarkably, genes related to transporters, DNA repair and recombination proteins, purine metabolism, ribosome, secretion systems, transcription factors, and pyrimidine metabolism were decreased in diarrheic piglets, but no significant biomarkers were found between groups using LEfSe analysis.

## Introduction

Diarrhea of neonatal piglets has long been a problem afflicting global piglets production. During the last few decades, reports have described diarrhea in neonatal pigs belonging to various age groups^1–3^. Porcine diarrhea leads directly to economic losses because of increased morbidity and mortality, reduced average daily gain (ADG), and the consumption of extra medication^4,5^. Intestinal microbes have a profound impact on health and disease through programming of immune and metabolic pathways^6^. Diarrhea has various causes, including porcine parvovirus, porcine kobuvirus, and enterotoxigenic *Escherichia coli* (ETEC)^7–10^, all of which have been linked to imbalances of normal intestinal flora as well as extra-intestinal microecological imbalance^11–13^. A number of recent studies have utilized high-throughput sequencing of the 16S rRNA gene to characterize gut microbiota of diarrheic piglets. Neonatal piglet diarrhea was associated with increases in the relative abundance of *Prevotella* (*Bacteroidetes*), *Sutterella* and *Campylobacter* (*Proteobacteria*)^14^. The percentage of *Enterococcus* (*Firmicutes*) was also more abundant in new neonatal porcine diarrhea (NNPD) affected piglets^13^. Genus *Veillonella* (*Firmicutes*) was the dominant bacteria in fecal microbiota in porcine epidemic diarrhea virus (PEDV)-infected piglets during the suckling transition stage^15^, while higher *Escherichia-Shigella* (*Proteobacteria*) in the feces was in Enterotoxigenic *Escherichia coli*-induced diarrhea in piglets^16^. Although Holman and the colleagues used a meta-analysis to define a “core” microbiota in the swine gut^17^, the key microbial populations related to diarrhea in piglets being poorly understood. Thus, we conducted a survey of porcine diarrhea in three medium-scale pig farms in Southwest China to investigate the effects of diarrhea on fecal microbiota. The cause of diarrhea was not considered when sampling, and a total of 52 and 33 swab samples were collected from diarrheic piglets and non-diarrheic piglets, respectively, of the same or similar age in the same hog house for 16S ribosomal RNA gene V4 region sequencing using the Illumina Miseq platform. We then compared and analyzed bacterial changes in the composition and function of the feces of piglets that were suffering from diarrhea and those that did not develop diarrhea to identify key differences in the fecal microbiota of piglets to reveal diarrhea-related bacteria.

## Results

### Overall information regarding the fecal microbiota of piglets

No significant differences in gender or sample location were discerned between diarrheic and non-diarrheic groups (*P* > 0.05; Table 1). Illumina Miseq sequencing of the V4 region of the bacterial 16S rRNA gene generated 6,868,150 high-quality sequences. After removal of chimeras, filtered high-quality sequences were grouped into 75,943 OTUs based on 97% species similarity (detail information of OTUs was shown in Supplementary Table 1).

**Table 1:** Overall microbiological and gene sequencing information regarding stool samples in this study.

Information	Group		*P* value
	Diarrheic (D)	Non-diarrheic (ND)	
Gender	29 (Female), 23 (Male)	24 (Female), 9 (Male)	0.12
Sampling location	16 (Sichuan), 36 (Chongqing)	15 (Sichuan), 18 (Chongqing)	0.22
Number of samples	52	33	
Average age	15 days-old	23 days-old	
Clean reads	76,206.58 ± 14,461.35	79,481.82 ± 12,609.14	
OTU	879.88 ± 343.35	914.82 ± 368.90	

Pairwise comparisons between groups were detected and values at *P* = 0.001, representing the grouping (D group and ND group), were valid. The four most abundant phyla in the fecal microbiota of diarrheic and non-diarrheic piglets were *Firmicutes*, *Proteobacteria*, *Bacteroidetes*, and *Fusobacteria* (Table 2). *Firmicutes* and *Bacteroidetes* constituted the top two phyla in the piglet gut microbiota in the ND group, whereas *Firmicutes* and *Proteobacteria* constituted the two predominant phyla in the gut microbiota of diarrheic piglets (D group). A similar abundance of *Firmicutes* was shown in the gut microbiota of piglets in groups D and ND (42.06% vs. 43.09%, *P* > 0.05). Diarrheic piglets showed a significantly lower percentage of *Bacteroidetes* and a higher percentage of *Proteobacteria* than non-diarrheic individuals (*P* < 0.05). Moreover, the *Proteobacteria-Bacteroidetes* ratio in the diarrheic group was 1.96, whereas the ratio in the non-diarrheic group was 0.36 (on average, Table 2).

**Table 2:** Percentage of the top four phyla in the gut microbiota of piglets in the diarrheic group (D group) and the non-diarrheic group (ND group).

Group	*Firmicutes*	*Proteobacteria*	*Bacteroidetes*	*Fusobacteria*
D group	42.06 ± 18.37	32.78 ± 28.21	16.75 ± 17.75	6.31 ± 8.24
ND group	43.09 ± 10.42	11.20 ± 9.69	31.53 ± 8.39	6.64 ± 5.28
P value	0.74	0.00	0.00	0.82
Cluster^1^				
Cluster A	40.24 ± 20.03	56.68 ± 19.54	1.24 ± 0.81	1.38 ± 3.46
Cluster B	52.37 ± 12.93	5.49 ± 2.21	32.44 ± 11.72	7.74 ± 4.85
Cluster C	43.08 ± 11.19	12.27 ± 10.91	30.92 ± 8.50	5.84 ± 5.10
Cluster D	43.12 ± 8.19	7.86 ± 2.08	33.44 ± 8.30	9.12 ± 5.43

^1^The experimental piglets used in the present study were early-weaned at 21 days of age. To evaluate overall differences in beta-diversity, we used principal coordinate analysis (PCoA) to identify discrepancies between groups. As shown in Fig. 1A, four distinct clusters were evident (Clusters A–D), and the relative abundance of the top four phyla in piglet microbiota were calculated.

The OTUs were also used to compare the differences in abundance between D and ND piglets (Table 3). The total abundance of 2 families, 11 genera, and 8 species differed significantly in the gut microbiota of D and ND piglets. For example, levels of the genera *Bacteroides*, *Ruminococcaceae*, and *Prevotella* in the fecal microbiota of diarrheic piglets were significantly lower than those in non-diarrheic piglets (*P* < 0.05). Diarrheic piglets also contained a significantly higher percentage of several species in the phylum *Proteobacteria*, including *Pasteurella aerogenes*, *Enterococcus cecorum*, *Enterococcus durans*, and *Escherichia coli* (*P* < 0.05).

**Table 3:** Comparison of the relative abundance of OTUs in the gut microbiota of D and ND piglets.

Taxonomic name^1^	Average%		*P* value	Tendency in diarrheic piglets compared with non-diarrheic samples
	D piglets	ND piglets		
Family				
*Clostridiales vadinBB60 group*	0.514%	2.220%	0.003	↓
*Erysipelotrichaceae*	0.780%	1.802%	0.018	↓
Genus				
*Allisonella*	0.994%	1.465%	0.033	↓
*Lactobacillus*	1.674%	0.393%	0.013	↑
*Bacteroides*	0.841%	1.705%	0.000	↓
*Ruminococcaceae NK4A214 group*	0.776%	1.807%	0.009	↓
*Ruminococcaceae UCG-002*	0.532%	2.193%	0.000	↓
*Ruminiclostridium 9*	0.823%	1.726%	0.000	↓
*Anaerotruncus*	0.637%	2.026%	0.000	↓
*Eubacterium coprostanoligenes group*	0.659%	1.993%	0.007	↓
*Family XIII AD3011 group*	0.750%	1.849%	0.000	↓
*Prevotella2*	0.789%	1.787%	0.005	↓
*Prevotella9*	0.849%	1.692%	0.015	↓
Species				
*Lactobacillus salivarius*	1.478%	0.708%	0.002	↑
*Lactobacillus vaginalis*	1.702%	0.349%	0.001	↑
*Lactobacillus gasseri*	0.445%	2.330%	0.020	↓
*Lactobacillus amylovorus*	1.588%	0.529%	0.003	↑
*Pasteurella aerogenes*	1.667%	0.404%	0.013	↑
*Enterococcus cecorum*	1.685%	0.381%	0.010	↑
*Enterococcus durans*	1.699%	0.353%	0.019	↑
*Escherichia coli*	1.670%	0.399%	0.000	↑

^1^OTUs for which the overall number in each sample was greater than 1000 and the number in half of the samples was greater than 100 were used to compare differences in abundances between D and ND piglets.

### Major microbial differences in different stages of piglet diarrhea

**Figure 1:**
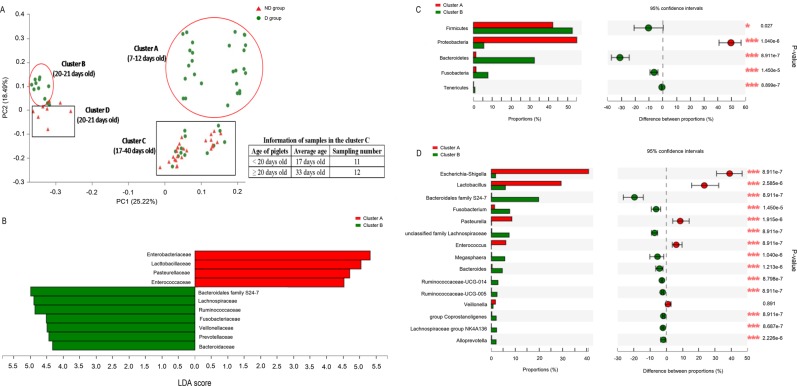
Comparison of fecal microbiota between diarrheic and non-diarrheic piglets. **(A)** Principal coordinate analysis (PCoA) shows the fecal microbiota of diarrheic (**D**) and non-diarrheic (ND) piglets. Red triangles, ND; green dots, D. **(B)** Identification of bacterial biomarkers in the fecal microbiota of diarrheic piglets in cluster A and cluster B using LEfSe analysis, and LDA scores >4.0. Comparison of the top four bacterial phyla **(C)** and the top fifteen bacteria genera **(D)** in the fecal microbiota of diarrheic piglets indifferent stages of development based on the Wilcoxon-rank-sum test are shown in the box plot (Cluster A: 7–12 day-old piglets; Cluster B: 20–21-day-old piglets). Samples in cluster A are in red, samples in cluster B are in green; *P < 0.05, ^**^P < 0.01, ^***^P < 0.001.

The experimental piglets used in the present study were early-weaned at 21 days of age. To evaluate overall differences in beta-diversity, we used principal coordinate analysis (PCoA) to identify discrepancies between groups. As shown in Fig. 1A, four distinct clusters were evident (Clusters A–D). The fecal microbiota of the ND group was distinct from that of group D, and the fecal microbiota of diarrheic piglets was distinct from the feeding phases. Specifically, the gut microbiota of 14 piglets (ranging in age from 7–12 days old) was gathered in cluster A, and these piglets were still in their lactation period. Cluster B contained the gut microbiota of 23 piglets (ranging in age from 20–21 days old) that were in the early weaning period. Cluster A was clearly differentiated from cluster B (Fig. 1A). Moreover, 52.17% of samples in cluster C were from piglets in the post-weaning period (average age = 33 days), and the gut microbiota of the D and ND piglets were indistinguishable, suggesting that the beta-diversity of their gut microbiota tended to be more similar across groups with age.

We used LEfSe analysis to identify biomarkers of fecal microbiota of diarrheic piglets (Fig. 1B) and found that the family *Enterobacteriaceae* was a biomarker of diarrheic piglets in cluster A (7–12 days old), whereas the *Bacteroidales* family *S24–7* was found to be a biomarker of diarrheic pigs in cluster B (20–21 days old). The Wilcoxon-rank-sum test was used to identify bacterial genera with significant differences in relative abundance in the fecal microbiota diarrheic piglets between clusters A and B. As shown in Fig. 1C,D, the genus *Escherichia-Shigella* in the family *Enterobacteriacae* was most abundant in cluster A, whereas the uncultured genus in the *Bacteroidales* family *S24–7* was the biomarker for cluster B.

### Core bacterial genera by co-occurrence network analysis

**Figure 2:**
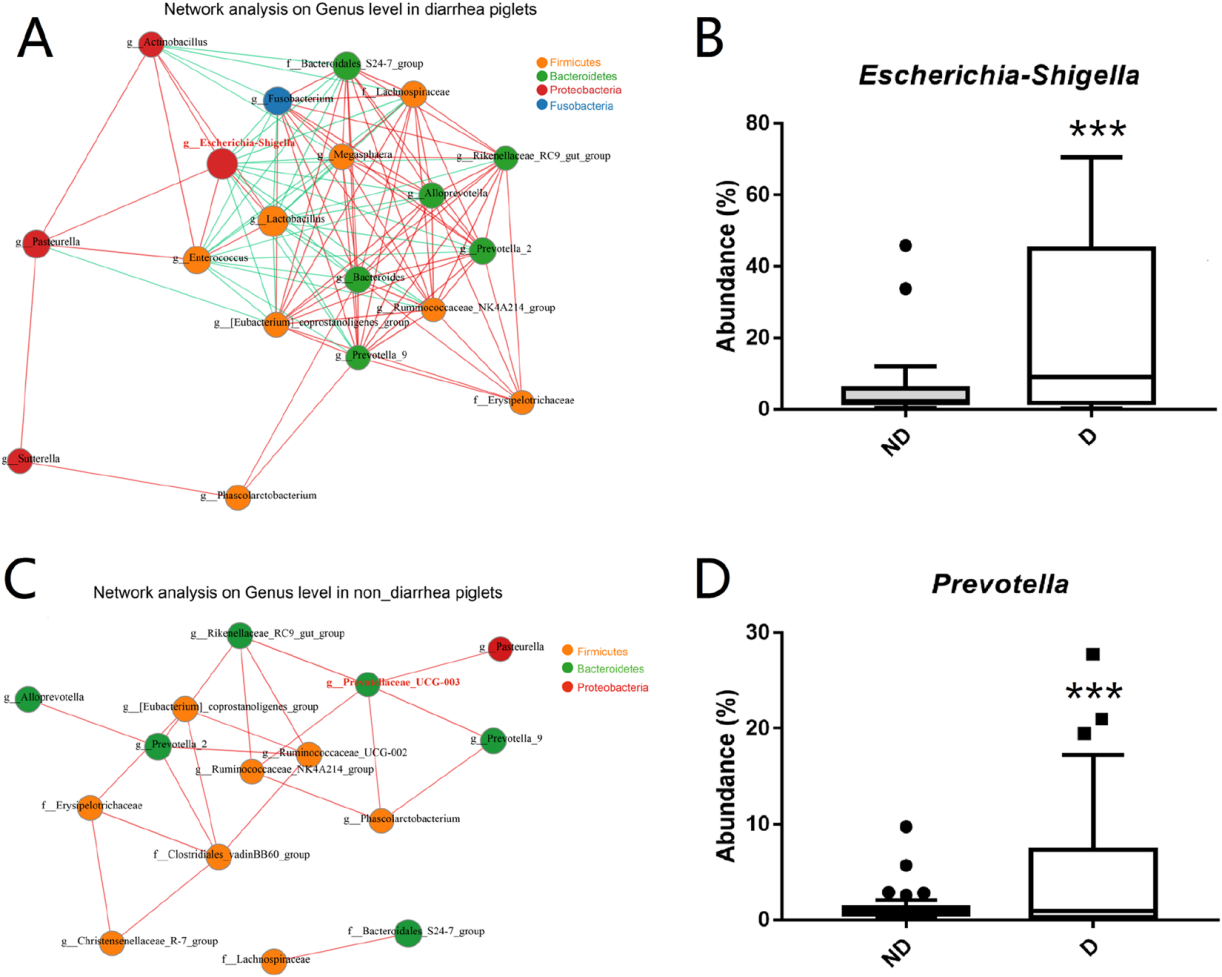
Co-correlation network analysis of bacterial genera constructed in diarrheic and non-diarrheic piglets. Co-correlation networks were deduced from the top 20 genera identified upon16S rRNA sequencing. Each node represents a genus, the size of each node is proportional to the relative abundance and the color of the nodes indicates their taxonomic assignment. The width of the lines indicates the correlation magnitude, while red represents a positive correlation and green a negative correlation. Only lines corresponding to correlations with a magnitude greater than 0.5 are shown. Co-correlation network of **(A)** *Escherichia-Shigella* genus in the diarrheic group (clustering = 0.82, closeness centrality = 0.86) and **(C)** *Prevotellaceae UCG-003* genus in the non-diarrheic group (clustering = 0.30, closeness centrality = 0.34). Comparison of the relative abundance of **(B)** the genus *Escherichia-Shigella* and **(D)** the genus *Prevotellaceae UCG-003* between diarrheic and non-diarrheic samples, which were visualized based on the means ± SEM. An independent *t* test was used to identify differences between groups. ^*^P < 0.05; ^**^P < 0.01; ^***^P < 0.001.

To identify the potential interactions that occur in response to diarrhea, co-correlative network analysis of the top 20 taxa was conducted for diarrheic and non-diarrheic piglets based on Spearman’s correlation coefficient (Fig. 2). Interestingly, we found that the genus *Escherichia-Shigella* was the core node in diarrheic samples, and that it tended to be positively correlated with aerobes and facultative anaerobes, such as the genera *Actinobacillus*, *Pasteurella*, *Enterococcus*, and *Lactobacillus*; however, it was negatively correlated with anaerobes, including the genera *Fusobacterium*, *Eubacterium coprostanoligenes group*, *Prevotella 2*, *Prevotella 9*, *Lachnospira*, *Rumniococcaceae NK4A214 group*, *Rikenellaceae RC9 gut group*, and *Alloprevotella* (Fig. 2A). The genus *Prevotellaceae UCG-003* was the core node in non-diarrheic piglets, and only positive correlations were found between *Prevotellaceae UCG-003* and anaerobes and facultative anaerobes, including the genera *Pasteurella*, *Prevotella*, *Phascolarctobacterium*, *Ruminococcaceae UCG-002*, and *Rikenellaceae RC9 gut group* (Fig. 2C). Among these marker genera, diarrheic samples comprised a significantly higher percentage of *Escherichia-Shigella* (22.92% vs.5.73%, *P* < 0.05), whereas non-diarrheic piglets contained a higher percentage of *Prevotella* (4.50% vs. 1.44%, *P* < 0.05) (Fig. 2B,D). The different core genera and the transition from negative correlations in diarrheic samples to positive correlations in non-diarrheic samples appeared to indicate that there was a correlation between bacterial competition for oxygen and the intestinal health of piglets.

We also found that members of the phylum *Proteobacteria* were reduced from four genera (*Escherichia-Shigella*, *Actinobacillus*, *Pasteurella*, and *Sutterella*) in the diarrheic group to only one genus (*Pasteurella*) in the non-diarrheic group, suggesting that an increase in the abundance and diversity of the phylum *Proteobacteria* played a pivotal role in piglet diarrhea.

### KEGG pathway analysis

**Figure 3:**
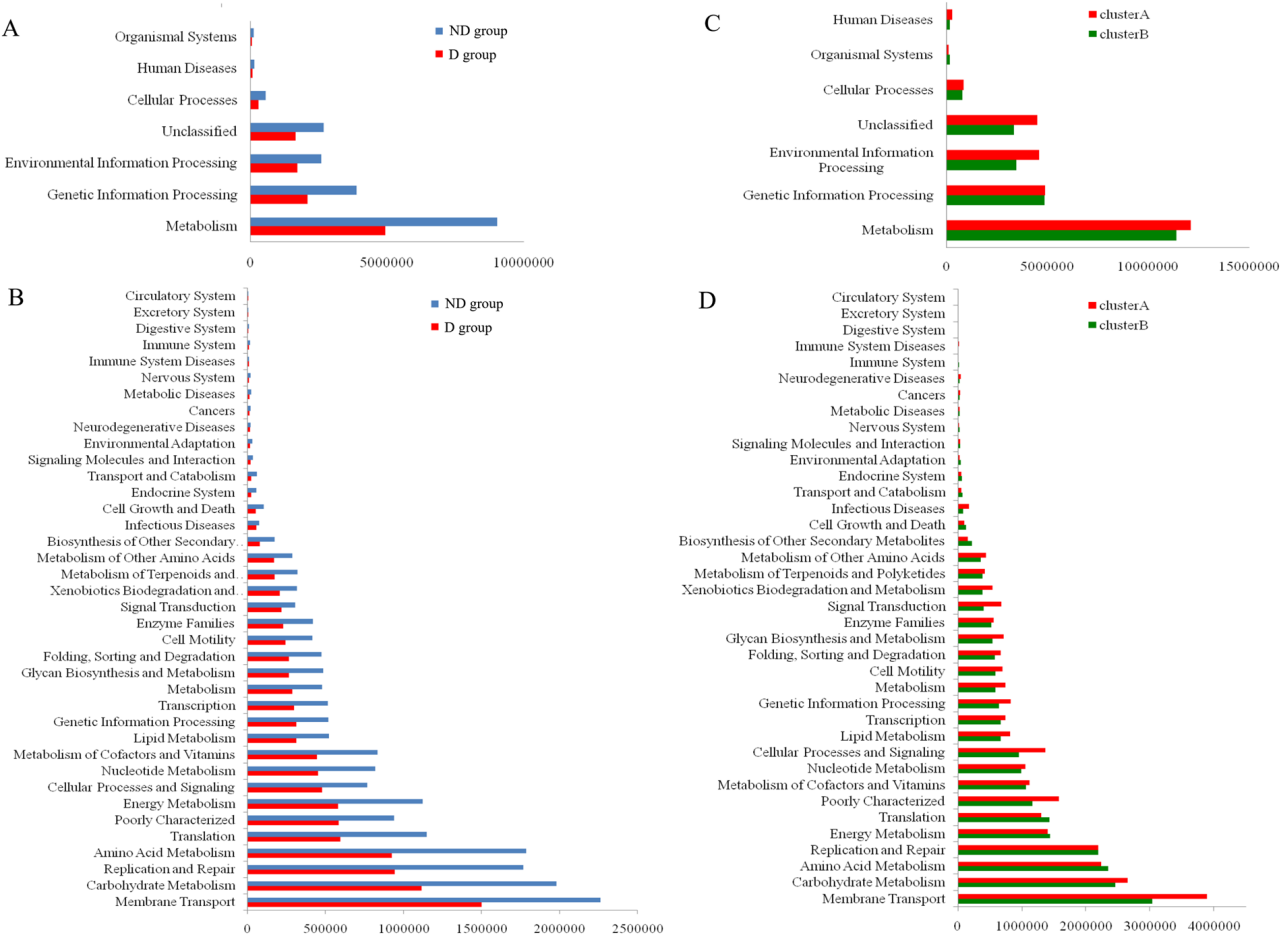
Comparison of variations in abundance of known KEGG pathways. The functional contributions of the gut microbiota were assessed using the PICRUSt tool. **(A,C)** Pathways at level 1 were obtained; **(B,D)** Pathways at level 2 were obtained. Diarrheic group (D group); non-diarrheic group (ND group); Cluster A: 7–12 day-old piglets; Cluster B: 20–21-day-old piglets.

To determine if enrichment of gut microbiota was associated with enrichment of specific metabolic activity associated with piglet diarrhea, the functional contributions of the gut microbiota were assessed using the PICRUSt tool. We found that KEGG pathways involved in membrane transport, carbohydrate metabolism, amino acid metabolism, and DNA replication and repair were predominant in both groups (Fig. 3A). Overall, 38 pathways related to membrane transport at level 2 were obtained, and membrane transport, carbohydrate metabolism, amino acid metabolism, and energy metabolism were major KEGG pathways in the fecal microbiota of piglets in this study (Fig. 3B). Interestingly, we also found that membrane transport was the most abundant pathway in the fecal microbiota of diarrheic piglets during lactation (cluster A) and weaning (cluster B) based on analysis of the functional contributions of the gut microbiota in cluster A and cluster B. We used LEfSe analysis to identify biomarkers of the KEGG pathways that differed significantly between diarrheic and non-diarrheic microbiota, as well as the number of significantly discriminative features with an LDA score >4.0. Similarly, no differentially abundant features of the KEGG pathways were found in the fecal microbiota of diarrheic piglets between cluster A and B (LDA score > 4.0). These findings clearly indicated that the occurrence of diarrhea in this study did not affect ecosystem processes of the fecal microbiota.

Moreover, we found that multiple KEGG (level 3) categories were disturbed when piglets had diarrhea. The gut microbiota of diarrheal piglets were characterized by a reduced representation of proteins involved in metabolism of pyrimidine and purine, transporters of the ATP-binding cassette, secretion systems as well as DNA repair and recombination (Table 4).

**Table 4:** The 10 most abundant KEGG pathways (at level 3) in the fecal microbiota of non-diarrheic and diarrheic piglets based on PICRUSt prediction.

Category Level 3	Diarrheic piglets (D group)	Non-diarrheic piglets (ND group)
Transporters	736755	1199406
General function prediction only	391357	688770
ABC transporters	377209	595634
DNA repair and recombination proteins	309864	562249
Purine metabolism	255530	443130
Ribosome	236991	481353
Two-component system	202776	279138
Secretion system	201378	242878
Transcription factors	199787	293331
Pyrimidine metabolism	194493	373833

## Discussion

Our study investigated variations in the composition and function of fecal microbiota between diarrheic piglets and non-diarrheic piglets. Consistent with the results of previous studies, *Firmicutes* was the dominant phylum in the piglet gut microbiota^18–20^, and there were no significant differences in relative abundance between groups (*P* > 0.05). *Proteobacteria* constituted the second most common phylum in the gut microbiota of diarrheic piglets, whereas *Bacteroidetes* was the second most abundant phylum in the fecal microbiota of non-diarrheic piglets (Fig. 1A and Table 2). When compared with non-diarrheic piglets, the abundance of the phylum *Proteobacteria* was significantly higher in samples from diarrheic piglets, while that of the phylum *Bacteroidetes* decreased significantly. Analysis of variations in bacterial genera between groups indicated that the genera *Prevotella* and *Ruminococcus*, which are known to be ubiquitous in the fecal microbiota of piglets^17^, were significantly lower in diarrheic samples (Fig. 1B and Table 3). Moreover, opportunistic bacteria in the phylum *Proteobacteria*^21^, including *Escherichia coli*^22^, *Pasteurella aerogenes*^23^, *Enterococcus cecorum*^24,25^, and *Enterococcus durans*^24–26^, were significantly higher in fecal samples from diarrheic piglets.

In the present study, we ignored the cause of piglet diarrhea, and instead focused on differences in the composition of fecal microbiota between groups. Surprisingly, beta-diversity analysis revealed that the fecal microbiota of diarrheic piglets was also differentiated by growth phases. Since piglets used in this study were early-weaned at 21 days of age, those aged less than 2 weeks were still in lactation. When combined with LEfSe analysis, the family *Enterobacteriaceae* was identified as a biomarker in diarrhetic piglets during lactation (from 7–12 days old in this study). An increase in *Proteobacteria* was previously reported as a marker for intestinal microbial community dysbiosis and a potential diagnostic criterion for disease^21^. A wide variety of opportunistic pathogens that belong to *Proteobacteria* are facultative anaerobes, and changes in the abundance of *Proteobacteria* might influence oxygen homeostasis or concentration in the gut^27^. Enrichment of *Proteobacteria*, such as *Enterobacteriaceae*, has also been observed in response to imbalances in the intestinal community and changes in animal health^28,29^.

The abundance of *Escherichia-Shigella* has been reported to decrease sharply as piglets mature from the suckling period to the weaning period^19^. Several species of *Escherichia* have been reported to be important to piglet diarrhea and to have a severe impact on animal intestinal barrier function^30,31^. Interestingly, in this study, a significant increase in *Escherichia*-*Shigella* that belong to the family *Enterobacteriaceae* was shown in microbial community of diarrheic piglets (Fig. 1D), which was assigned as the core node in diarrheic piglets (Fig. 2). *Prevotellacecea UCG-003* was identified as a key node in non-diarrheic piglets upon co-correlation network analysis, and differences in the core genus and the transition from negative correlations in diarrheic samples to positive correlations in non-diarrheic samples indicate that there is a correlation between bacterial competition for oxygen and the intestinal health of piglets.

In this study, the average abundance of the *Bacteroidales* family *S24–7* and *Escherichia-Shigella* in diarrheic piglets (D group) was 4.94% and 24.50%, whereas their average abundance in non-diarrheic piglets (ND group) was 7.41% and 5.99%, respectively. This change in fecal microbiota reflected the different causes of swine diarrhea in different stages after birth. One important reason for piglet diarrhea in lactation in this study was the expansion of swine enteric pathogens (e.g., *Escherichia-Shigella*). However, when grown, the average abundance of *Escherichia-Shigella* in the gut microbiota of diarrheic piglets during weaning was only 1.80% (cluster B, shown in Fig. 1D), suggesting that these enteric pathogens were weakly correlated with diarrhea in weaning pigs in this study. Abrupt changes in the diet and environment of piglets have been reported as the leading causes of weaning diarrhea^32,33^. Interestingly, there was an enormous increase in members of the fiber-degrader *Bacteroidales* family *S24–7*^34,35^ when piglets grew up (less than 1.00% in cluster A versus 20.04% in cluster B). However, very little work regarding *Bacteroidales* family *S24–7* has been conducted to date. In short, it is necessary to conduct ongoing research regarding its biological function and usage. Nevertheless, the above results suggest that the focus of early weaning syndrome in piglets should be shifted from intestinal pathogens to moderate changes in diet and better feeding and management.

In the present study, we also found a dysbiosis of intestinal microbiota in diarrheic samples, especially the higher percentage of several *Lactobacillus* strains, which was consistent with the results of a previous study^36^. The increased abundance of the GABA-producing *Lactococcus lactis* led to increased expression of IL-17 during piglet ETEC infection^37^. In the present study, several *Lactobacillus* strains, including *Lactobacillus salivarius*, *Lactobacillus vaginalis*, and *Lactobacillus amylovorus*, were higher in the diarrheic microbiota (Table 3). *Lactobacillus salivarius* is known for its ability to produce lactic acid. In addition to lactic acids, *Lactobacillus salivarius* also produced γ-aminobutyric acid^38^. Similar to *Lactococcus lactis*, we believe that this GABA-producing strain may have increased GABA signaling to actively affect host health and disease states. Future studies should be conducted to investigate this and explore the mechanisms responsible for the increased abundance of specific *Lactobacillus* strain(s) during piglet diarrhea.

The ABC transporters are primary transporters that couple the energy stored in adenosine triphosphate (ATP) to the movement of molecules across the membrane, which link with multi-drug resistance in both bacteria and eukaryotes^39^. A general overview of the DNA damage response pathway in humans indicated that deficient DNA repair could affect genome stability, which could induce tumorigenesis^40^. In this study, PICRUSt prediction revealed that the relative abundance of ABC transporters, DNA repair, and recombination proteins were downregulated in the fecal microbiota of diarrheic piglets, implying multi-drug resistance and DNA in swine cells was damaged when diarrhea occurred. However, no differentially abundant KEGG pathways were found in the fecal microbiota of diarrheic and non-diarrheic piglets with a LDA score >4.0 (Fig. 3). A reliable reason for why changes in microbial composition did not affect their functional contributions is that the taxa in the microbial community of diarrheic piglets were functionally redundant^41^ with the taxa in the community of non-diarrheic piglets.

## Conclusion

We revealed the main variations in the composition of fecal microbiota of diarrheic piglets and non-diarrheic piglets. *Proteobacteria* was the second most abundant phylum in intestinal microbiota of diarrheic piglets. We found that the fecal microbiota of diarrheic piglets was differentiated by animal growth phases, and the family *Enterobacteriaceae* was a biomarker in piglets during lactation, but the *Bacteroidales* family *S24–7* group was a biomarker in later stages of growth. In addition, *Escherichia-Shigella* was the core in diarrheic gut microbiota, whereas *Provteollaceace UCG-003* was the core in the fecal microbiota of non-diarrheic piglets.

## Materials and Methods

### Ethics statement

All animal experiments were conducted pursuant to the Regulations for the Administration of Affairs Concerning Experimental Animals (Ministry of Science and Technology, Beijing, China, revised June 2014). All guidelines related to the care of laboratory animals were followed. The institutional ethics committee of the Chongqing Academy of Animal Sciences (Chongqing, China) reviewed the relevant ethical issues and approved this study (permit number xky-20150113). Only fresh stool samples collected by rectal swabs were analyzed, and no animals were killed or injured in this study. The preparation of total genomic DNA was conducted at the Experimental Swine Engineering Center of the Chongqing Academy of Animal Sciences (CMA No. 162221340234; Rongchang, Chongqing, China).

### Sample collection

In the present study, piglets were early-weaned at 21 days of age. We collected a total of 85 piglet fecal samples during January of 2016. Specifically, 31 samples were collected from Shuangjia Farm (Longchang County, Sichuan Province, China), 41 were obtained from Taoranju Farm (Rongchang District, Chongqing, China), and 13 were obtained from Pengkang Farm (Yongchuan District, Chongqing, China). Overall, 52 piglets had diarrhea (diarrhea group or D group), which was characterized by liquid, yellow-green or taupe feces with a foul smell or stench that stuck around the anus. Thirty-three piglets had no diarrhea (non-diarrhea group or ND group), as indicated by solid feces with no blood or mucus and no waste attached around the anal area (non-diarrhea group or ND group).

About 0.5 g of freshly passed stool from the swab samples was transferred into a sterile Eppendorf tube (Axygen Inc., Union City, CA, USA), after which 10% glycerol (vol/vol) in sterile pre-reduced saline was added to each tube. The samples were then homogenized and then immediately frozen at−80 °C until needed for 16S ribosomal RNA gene sequencing.

### Sequencing and Analysis

#### 16S rRNA gene sequencing

Total genomic DNA was extracted from samples using the CTAB/SDS method, after which the 16S rRNA gene of the distinct 16S V4 region was amplified using specific primers (515F–806 R) with a barcode. The microbial diversity and composition were then determined by 16S rRNA gene sequencing and analysis as previously described^6^.

#### LDA effect size (LEfSe)

To identify the genomic features of taxa differing in abundance between two or more biological conditions or classes, the LEfSe (Linear Discriminant Analysis Effect Size) algorithm was used with the online interface Galaxy (http://huttenhower.sph.harvard.edu/lefse/)^42^. A size-effect threshold of 4.0 on the logarithmic LDA score was used for discriminative functional biomarkers.

#### Co-correlation statistics

According to the calculation method developed by Hartmann *et al*.^43^, co-correlation networks were generated using the python package NetworkX (https://github.com/networkx/networkx) and the OTUs as target nodes, with edges (e.g., connecting nodes) representing significant negative (green) or positive (red) Spearman’s correlations. We retained OTUs when they had a Spearman’s correlation coefficient >0.5.

#### Predicted functionality of the differently grouped samples

Phylogenetic Investigation of Communities by Reconstruction of Unobserved States (PICRUSt) (http://galaxy.morganlangille.com/)^44^ was used to predict the functional gene content in the fecal microbiota based on taxonomy from the Greengenes reference database (http://greengenes.lbl.gov/cgi-bin/nph-index.cgi). First, a collection of closed-reference OTUs was obtained from the filtered reads using QIIME (v 1.7.0, http://qiime.org/scripts/split_libraries_fastq.html)^45^, and by querying the data against a reference collection (Greengenes), after which OTUs were assigned at 97% identity. The resulting OTUs were then employed for microbial community metagenome prediction with PICRUSt using the online Galaxy interface (http://huttenhower.sph.harvard.edu/galaxy/). Supervised analysis was conducted using LEfSe to elicit the microbial functional pathways that were differentially expressed among samples. PICRUSt was used to derive relative Kyoto Encyclopedia of Genes and Genomes (KEGG) Pathway abundance.

### Statistical analysis

Data proportions of sites and gender were regarded as categorical variables and compared by the Chi-square test. Pairwise comparisons between groups were assessed by analysis of similarity (ANOSIM). Values represent the pairwise test statistic (R) for ANOSIM. The permutation-based level of significance was adjusted for multiple comparisons using the Benjamini-Hochberg false discovery rate (FDR) procedure. A *P* < 0.05 indicates the difference between groups is greater than the difference within the group. The Wilcoxon-rank-sum test was used to detect the different populated bacterial genera between groups. The relative abundances of bacterial taxa are presented as the means ± SD, and differences between groups were identified by the independent-sample *t* test (for normally distributed data) or the Mann-Whitney U-test (for non-normally distributed data). A *p*-value <0.05 was considered statistically significant, and a *p*-value <0.01 indicated extreme significance. The raw sequences obtained in the present study have been submitted to the NCBI Sequence Read Archive (accession number SRP134239).

## Supplementary information

Supplementary Table 1
